# The effects of oviposition-site deprivation on longevity and bloodfeeding rate in *Anopheles gambiae*

**DOI:** 10.1186/1756-3305-7-163

**Published:** 2014-04-03

**Authors:** Monica L Artis, Diana L Huestis, Tovi Lehmann

**Affiliations:** 1 Laboratory of Malaria and Vector Research, National Institutes of Health, Bethesda, MD, USA

**Keywords:** Aestivation, African malaria mosquito, Dry season, Drought, Vectorial capacity

## Abstract

**Background:**

The African malaria mosquito, *Anopheles gambiae*, needs surface water in order to lay their eggs. In many parts of Africa, there are dry periods varying from days to months in length when suitable larval sites are not available and female mosquitoes experience oviposition-site deprivation (OSD). Previous studies have shown that egg-laying and egg-hatching rates were reduced due to OSD. Here, we assessed its effect on longevity and bloodfeeding rate of *Anopheles gambiae*. We predicted that OSD will increase mosquito longevity and the aptitude of mosquitoes to take additional blood meals; importantly, these changes will increase its vectorial capacity.

**Methods:**

To measure the effect of OSD, four treatments were utilized: two oviposition-deprived groups, one of which was bloodfed once (OBOD) and one that was bloodfed weekly (MBOD); a non-oviposition-deprived, weekly bloodfed control group (MBC); and a blood-deprived age-control group (BD). Mortality was assessed daily and bloodfeeding rate was measured at weekly intervals.

**Results:**

Under OSD, survival of female *A. gambiae* was reduced by 10-20%, reflecting reduction of the MBOD and OBOD groups from the MBC group, respectively. Likewise, bloodfeeding response during three weeks of OSD was reduced but the reduction varied as a function of time from the last blood meal.

**Conclusions:**

These results indicate that OSD is expected to reduce *A. gambiae* vectorial capacity and that OSD alone does not act as cue used by female mosquitoes to switch into a dormant state of extended survivorship with reproductive quiescence.

## Background

The African malaria mosquito, *Anopheles gambiae*, needs surface water in order to lay eggs. In many areas, larval sites are unavailable during short and long dry spells when mosquitoes endure oviposition-site deprivation (OSD). The effects of OSD on the mosquito and her capacity to transmit disease are poorly understood. In areas such as the Sahel, when no larval sites are available during the dry season for two to seven months, mosquitoes must ensure their survival until the resumption of rains [1-4]. Possibly, OSD may play a role in extending longevity by shifting mosquito physiology into a reproductively depressed state [5,6]. The longer a female mosquito survives, the higher the risk she poses in terms of malaria transmission [7-10]. Infection of the vector requires at least one bloodmeal followed by oviposition (gonotrophic cycle), and disease transmission further requires survival of the female for enough time to become infectious before she can inoculate the pathogen into a new host [10,11].

The effects of OSD on mosquito reproduction was previously assessed in *Aedes aegypti*[12,13], *Aedes sollicitans*[14], *Culex fatigans*[15], *Culex quinquefasciatus*[16], *Anopheles phareoensis*[17], and *Anopheles maculatus*[15]. These studies reported that *A. aegypti*[13,15] and *C. fatigans* were less affected by OSD than were *A. maculatus*[15] and *A. pharoensis*[17], yet they all exhibited depressed reproductive output. Similar to the other anophelines studied, *A. gambiae* was found to be sensitive to short-term OSD, since even one or two weeks of OSD dramatically reduced its egg-batch size and egg-hatching rate [5]. The longer the female was subjected to OSD, the greater these effects were [5]. The authors also found that supplemental bloodmeals diminished the effect of OSD by partially restoring both oviposition rate and embryonic development, resulting in a partially rescued hatch rate.

Despite its possible relevance for modulating vectorial capacity, to the best of our knowledge, the effect of OSD on female bloodfeeding rate and longevity has not been assessed in *A. gambiae* or in other mosquito species. We hypothesized that oviposition-deprived females, especially those with access to bloodmeals, would live longer than non-oviposition-deprived controls, because they could potentially allocate more nutrients into “maintenance” and avoid the direct and indirect costs of reproduction [18]. Additionally, OSD may act as a signal to shift mosquito physiology into short- or long-term dormancy geared to extend survival [1,2,6,19]. We also predicted that the oviposition-deprived females would take more frequent blood meals to lessen the impact of OSD, as suggested by Dieter and colleagues [5], and that multiply bloodfed OSD females would survive longer than both the non-deprived females (control) and once-bloodfed oviposition-deprived females. Here, we test these hypotheses in a laboratory colony of *A. gambiae*, extending our previous study [5].

## Methods

### Experimental design

**Figure 1:**
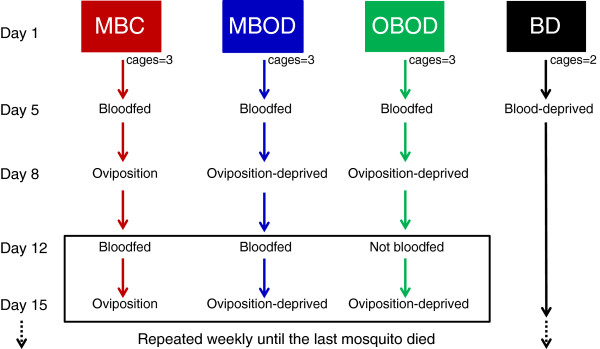
**Diagram of the experimental design used in this study.** Treatments are abbreviated as follows: multiple-bloodfed non-oviposition-deprived control group (MBC; red), multiple-bloodfed oviposition-deprived (MBOD; blue), once-bloodfed oviposition-deprived (OBOD; green), and blood-deprived age control (BD; black). 150 newly-emerged females were placed in each cage at the start of the experiment. The boxed area and dotted arrows indicate that these steps were repeated weekly until the last mosquito in the experiment died.

Mosquitoes from the NIH G3 *A. gambiae* colony were used for this experiment; this colony is adapted to feed on chickens and has been used in prior similar experiments [5]. Newly emerged male and female mosquitoes from a single large cohort were randomly divided into 11 cages, consisting of 150 of each sex, for a total of 3,300 individuals. Round plastic cages (22 cm diameter × 17.5 cm height) covered with a net top were used. The cages were randomly assigned to four different treatments: blood-deprived, normal bloodfeeding and oviposition, and two oviposition-site deprivation treatments with different bloodfeeding regimens (fed weekly and fed only once, detailed further below; Figure 1). When the mosquitoes were 5 days old, white leghorn chickens (*Gallus gallus*) were used to bloodfeed the mosquitoes assigned to the three bloodfed treatments. Mosquitoes were allowed to feed on the chicken for 15 minutes and were given an additional 5 minutes if the feeding rate was < 50%. The day after the first bloodfeeding, all unfed female mosquitoes were removed and the following day (7 days of age) males were removed, after measurements of insemination (by dissection of the spermatheca) indicated over 90% insemination (n = 25, overall cages). Throughout the experiment, all treatments were given sugar pads (10% Karo syrup) daily and mosquitoes were kept in the insectary under 12:12 L:D cycle, 27°C, and 75% RH.

Four treatments were used to assess the effects of OSD separate from the effects of age and bloodmeals (see Figure 1). In the once-bloodfed oviposition-deprived (OBOD) treatment, mosquitoes were bloodfed during the first feeding cycle and then sugar-fed for the rest of the experiment without receiving water for oviposition. The other OSD group received weekly bloodfeedings without receiving water for oviposition (multiple-bloodfed oviposition-deprived, hereafter MBOD). To simulate the normal mosquito life-cycle, we had a multiple-bloodfed non-oviposition-deprived control group (MBC) that received weekly bloodfeedings and water for oviposition. Finally, we had a blood-deprived (BD) age control treatment that did not receive bloodmeals or water for oviposition. After the initial bloodfeeding at 5 days of age, the MBOD and MBC groups were bloodfed every 7 days (see Figure 1). However, unfed mosquitoes were not removed subsequent to the first blood meal (above). Estimates of feeding rate by counting 30 mosquitoes in one sector of the cage were over 85%. In our insectary, the gonotrophic cycle of the G3 colony is four days long and it is routinely maintained by feeding on a weekly basis. To maximize oviposition and bloodfeeding receptivity, we provided egg dishes on days four and five post-feeding while blood-feeding was offered on a weekly basis.

To assess bloodfeeding rate, a weekly bloodfeeding assay was performed with 7 mosquitoes from each cage (excluding the BD) prior to each of the first four bloodfeedings. These mosquitoes were put into pint-size paper cups for 4 hours to adjust before they were given an opportunity to bloodfeed on a chicken for 5 minutes. The mosquitoes were killed by freezing and then smeared on white paper to check for the presence of blood; the number that bloodfed was recorded for each group. To measure mosquito longevity, dead mosquitoes were removed daily and preserved in tubes filled with 80% ethanol.

Wing length as a proxy of body size was measured after removing one wing from each specimen and mounting the wing in glycerol. Pictures of the wings were taken at 25× magnification using a Leica DM 4500B microscope and wing length was measured as previously described [20]. The wing-length measurements were obtained for one-quarter of the mosquitoes from each cage (approximately 32 per cage, totaling 354 mosquitoes). To eliminate selection bias by age, mosquitoes were sorted by longevity and every fourth specimen was selected for measurement.

### Statistical methods

Differences in body size (measured as wing length) among groups was evaluated using univariate ANOVA implemented by Proc GLM in SAS [21]. To assess the effect of body size on longevity and the heterogeneity of slopes reflecting treatment-specific size effects, we used an analysis of covariance (ANCOVA) by including group, body size, and their interaction, implemented by the same procedure. Differences in survival (longevity) were visualized and tested using a Wilcoxon test in Proc Lifetest [21]. Mosquitoes that were lost or removed from the longevity experiment and those subjected to the bloodfeeding assay (above) were treated as censored individuals. Calculation of the expected longevity incorporated censored mosquitoes. A statistically significant global test including all four treatments was followed by all unique pairwise tests (total of 6 tests). The sequential Bonferroni test [22] was then used to accommodate the number of tests in the decision regarding the significance of each individual test. Differences between treatments in bloodfeeding response were assessed after pooling the seven mosquitoes from different cages (within treatment) to increase the sample size to 21/treatment/assay. Here, we assume that variation between cages of the same treatment is negligible compared with that between treatments. A total of 4 assays were conducted at ages 5, 12, 19, and 26 d, reflecting pre-OSD and 1, 2, and 3 weeks of OSD, respectively. Each assay was subjected to a global test to determine if bloodfeeding rates were different across the three treatments using an exact test implemented by permutation of *χ*^2^ tests in Proc Freq (SAS). Only if the global test was significant (P < 0.05), three additional pairwise tests were performed to determine which treatments differed from each other using the same (exact) test. The sequential Bonferroni test [22] was then used to accommodate the number of tests in the decision regarding the significance of each individual test.

## Ethics approval

This study was carried out in accordance with the recommendations in the Guide for the Care and Use of Laboratory Animals of the National Institutes of Health. All animal procedures were approved by the National Institutes of Health Animal Care and Use Committee (ACUC, Protocol ID: LMVR102).

## Results and discussion

### Body size

Because body size is a known determinant of mosquito longevity [7,18,23], we evaluated the differences in body size between treatments and also its effect on longevity in the present experiment. Mean body size, measured by wing length, did not vary significantly among treatments (P < 0.6, F_3/347_ = 0.59; Table 1) or cages (P < 0.46, F_10/340_ = 0.97), as expected given the random assignment of individuals to cages (see Methods). Similar to previous studies [18], expected longevity increased by two days with an increase in 0.1 mm in wing length (P < 0.001, F_1/343_ = 22.01); however, no significant difference was found in slopes of the different treatments as the interaction between wing length and treatment was not statistically significant (P > 0.7, F_3/343_ = 0.4; Table 1). Because there were no differences in body size among treatments and no heterogeneity in the specific effect of body size on longevity between treatments, body size was excluded from further analyses comparing longevity between treatments (see below).

**Table 1:** Body-size variation within and between treatments and measures of the effect of body size on longevity

**Treatment**	**N**_**WL **_**(N**_**cages**_**)**	**Mean**_**WL **_**(SE**_**WL**_**)**	**Slope (intercept)**^**a**^	**P-value**^**a**^
Once-bloodfed oviposition-deprived (OBOD)	100 (3)	2.81 (0.017)	18.7 (-20.9)	0.008
Multiple-bloodfed oviposition-deprived (MBOD)	100 (8)	2.82 (0.018)	15.8 (-11.7)	0.008
Multiple-bloodfed non-oviposition-deprived control (MBC)	88 (3)	2.84 (0.022)	11.5 (3.7)	0.051
Blood-deprived age control (BD)	55 (2)	2.81 (0.023)	20.0 (-31.7)	<0.001

^a^Relationship between body size and longevity. Slopes were not significantly different between treatments (P>0.75; see text).

Note: Abbreviations are in Figures 1, 2 and 3.

### Longevity

**Figure 2:**
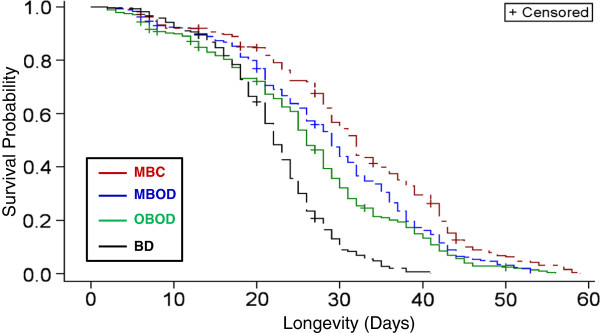
**Mosquito longevity over time in each treatment group.** Each survival function was found to be statistically different from all others (see text for details). Treatment abbreviations and colors are as in Figure 1.

Overall, adult longevity varied between 2 and 59 days. Differences in mean longevity among treatments were seen by 15 days of age and persisted thereafter (Figure 2). The median expected survival of mosquitoes from the different groups were 22 (range = 2-41), 26 (range = 2-56), 29 (range = 2-53), and 32 (range = 2-59) for the BD, OBOD, MBOD, and MBC groups, respectively; all were significantly different from each other after accommodating for multiple tests using the sequential Bonferroni test. Contrary to our hypothesis, OSD did not increase mosquito survival (Figure 2). In fact, mosquito survival of the MBC, representing the normal gonotrophic cycle, was the highest, although it was followed closely by the MBOD; the difference in expected mean survival of these groups was 10% (Figure 2). Mosquitoes in the BD group had the lowest mean survival (P < 0.001; Figure 2), as expected based on previous studies [18,24], highlighting the value of the bloodmeal not only for egg development in mosquitoes, but also for survival. Variation between cages in each treatment (separately) was tested using the same procedure. Differences among cages within groups were not significant (P > 0.087) in all treatments except OBOD (P = 0.048), however, because this too was insignificant by the sequential Bonferroni test, heterogeneity between cages was rejected. These results suggest that the physiological disruption of OSD, including aborted egg development and egg degradation [5] outweigh the direct and indirect benefits associated with the apparent surplus of nutritional reserves available for survival and the elimination of egg-laying. Possibly the allocation of resources in reproduction is not reversible or the reversion requires a high investment of resources. Alternatively, mosquitoes continue to search for a suitable oviposition site – a costly and long pursuit, consuming the resources needed for survival. We have not measured the flight activity of the females from the different groups, so we cannot evaluate this hypothesis. Availability of supplemental bloodmeals (MBOD), however, improved survival of mosquitoes subjected to OSD (OBOD), as shown in Figure 2.

These results also suggest that OSD by itself does not serve as a cue used by mosquitoes to switch their physiology from reproduction to long-term survival. It is consistent with the aestivation (dry-season diapause) hypothesis, which unlike quiescence, requires token environmental stimuli such as changing photoperiod to anticipate the coming hardship rather than responding to the hardship as it unfolds [25-28]. Accordingly, the presumably aestivating *A. coluzzii (*previously known as the M-form of *A. gambiae*) has been observed to nearly disappear from villages approximately one month before the larval sites disappear [1,2,6,19]. These results provide evidence that OSD does not act as the cue for dormancy or that quiescence is involved. However, it is possible that OSD may be important in maintaining the state of dormancy of females already dormant.

### Bloodfeeding rate

**Figure 3:**
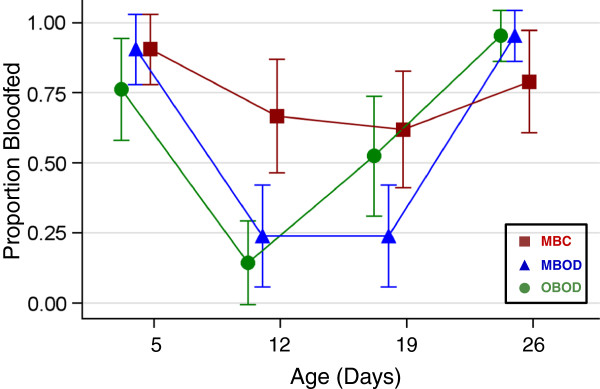
**Bloodfeeding rates of the feeding assay performed in the first 4 weeks (see Methods) by treatment group.** Mean and standard error of the replicates are represented. Abbreviations and colors of treatment groups as in Figure 1.

Weekly bloodfeeding assays were performed to evaluate the effect of short- and long-term OSD in relation to mosquito age. At 5 days of age, all groups exhibited similar and high feeding rates (near 90%; Figure 3), as expected, because the treatments had not yet taken effect. After one week of OSD (day 12), the two oviposition-deprived treatments exhibited a reduced feeding rate (~20%) compared with the MBC group (70%; P < 0.02, Exact test; Figure 3). As expected, there was no difference between the two OSD treatments at that time (P > 0.65, Exact test; Figure 3). The MBC group had a high and consistent feeding rate in the next two assays (days 19 and 26) that did not differ significantly across all four weeks that the feeding-assays were performed (range = 62-91%, P > 0.14, Exact test; Figure 3). However, there was a significant increase in the bloodfeeding response of the OBOD group after the second week of OSD (53%, P < 0.021, Exact test; Figure 3), likely due to females having already completed egg degradation. In contrast, blood and/or egg degradation may have continued in females of MBOD group, which exhibited low bloodfeeding response (24%, P > 0.5 between 2^nd^ and 3^rd^ feeding assay, Exact test; Figure 3). By the fourth week, all three treatments were again relatively high and not significantly different from one another (P > 0.18, Exact test; Figure 3). Overall, these results suggest that bloodfeeding rates in mosquitoes subjected to OSD is reduced compared with non-deprived females that undergo normal gonotrophic cycles. The differences between groups subjected to multiple bloodfeedings as opposed to one bloodfeeding may have been diminished because not all mosquitoes that were offered a bloodmeal actually fed each week.

Oviposition-site deprivation was shown to reduce egg-batch size and hatch rate in *A. aegypti*, *A. albopictus*, *C. quinquefasciatus*, *A. maculatus*, *A. pharoensis*, and *A. gambiae*[5,12,14-17]. The effects of OSD were manifested as early as 2 days (*A. pharoensis*) and 7 days (*A. gambiae*) to as high as 70 days (*A. albopictus* and *C. quinquefasciatus*). Thus, anopheline species appear more sensitive to shorter-term OSD as measured by their egg-batch size and hatch rate. The lack of oviposition sites can cause mosquitoes to retain and reabsorb their eggs [29]. Partly degraded eggs were observed in our previous study [5], although additional bloodmeals diminished the reduction in egg-batch size and hatch rate, leading to the hypothesis that bloodfeeding rate is elevated in females subjected to OSD. Contrary to this hypothesis, the present results suggest that bloodfeeding rate was reduced.

Degradation of eggs appears to be a very costly process in *A. gambiae*, using nearly all of the resources that were invested in egg development or reducing survivorship irrespective of the available resources (e.g., elevated oxidative stress), because supplemental blood meals had limited improvements on survivorship (present study) or reproduction success [5]. Whether species and populations inhabiting areas subjected to frequent or rare dry spells vary in their response is of much interest and cannot be inferred from a study on a laboratory colony.

## Conclusions

Because the range of *A. gambiae s.l.* includes dry regions where short and long dry spells are common, its high sensitivity to short-term OSD is surprising. Here, we extend previous studies showing that 7–14 days OSD dramatically reduce *A. gambiae*’s reproductive output and show that its sensitivity to OSD includes reduced longevity and reduced bloodfeeding rates. It is therefore concluded that vectorial capacity of *A. gambiae* is diminished by OSD. Further, OSD alone probably does not act as a cue to switch its physiology from maximizing reproductive output to maximizing survival. However, our experiments were conducted with a laboratory-adapted colony of tropical origin (G3) and may not reflect the responses of natural mosquitoes from populations that experience extended OSD on a regular basis, such as many Sahelian populations of *A. coluzzii* and Sudanese *Anopheles arabiensis*. Thus, additional work is required to assess variation in the response to OSD in populations that frequently experience short-term OSD as well as in those that experience extended OSD regularly.

## Competing interests

The authors declare that they have no competing interests.

## Authors’ contributions

The experiment was designed by MLA, DLH, and TL. Data were collected by MLA and DLH and analyzed primarily by TL. The manuscript was drafted by TL and MLA and edited by DLH. All authors read and approved the final manuscript.
